# Associations between polygenic risk for schizophrenia and brain function during probabilistic learning in healthy individuals

**DOI:** 10.1002/hbm.23044

**Published:** 2015-10-28

**Authors:** Thomas M. Lancaster, Niklas Ihssen, Lisa M. Brindley, Katherine E. Tansey, Kiran Mantripragada, Michael C. O'Donovan, Michael J. Owen, David E.J. Linden

**Affiliations:** ^1^ Neuroscience and Mental Health Research Institute, Cardiff University Cardiff United Kingdom; ^2^ School of Psychology Cardiff University, Cardiff University Brain Research Imaging Centre (CUBRIC) 70 Park Place, Cardiff, CF10 3AT Wales United Kingdom; ^3^ Cardiff School of Medicine Cardiff University, MRC Centre for Neuropsychiatric Genetics and Genomics, Institute of Psychological Medicine and Clinical Neurosciences Cardiff United Kingdom

**Keywords:** fMRI, polygenic, schizophrenia, reward, reversal learning

## Abstract

A substantial proportion of schizophrenia liability can be explained by additive genetic factors. Risk profile scores (RPS) directly index risk using a summated total of common risk variants weighted by their effect. Previous studies suggest that schizophrenia RPS predict alterations to neural networks that support working memory and verbal fluency. In this study, we apply schizophrenia RPS to fMRI data to elucidate the effects of polygenic risk on functional brain networks during a probabilistic‐learning neuroimaging paradigm. The neural networks recruited during this paradigm have previously been shown to be altered to unmedicated schizophrenia patients and relatives of schizophrenia patients, which may reflect genetic susceptibility. We created schizophrenia RPS using summary data from the Psychiatric Genetic Consortium (Schizophrenia Working Group) for 83 healthy individuals and explore associations between schizophrenia RPS and blood oxygen level dependency (BOLD) during periods of choice behavior (switch–stay) and reflection upon choice outcome (reward–punishment). We show that schizophrenia RPS is associated with alterations in the frontal pole (*P*_WHOLE‐BRAIN‐CORRECTED_ = 0.048) and the ventral striatum (*P*_ROI‐CORRECTED _= 0.036), during choice behavior, but not choice outcome. We suggest that the common risk variants that increase susceptibility to schizophrenia can be associated with alterations in the neural circuitry that support the processing of changing reward contingencies. *Hum Brain Mapp 37:491–500, 2016*. © **2015 Wiley Periodicals, Inc.**

## INTRODUCTION

Schizophrenia is highly heritable, with recent genome‐wide association studies (GWAS) suggesting that over one hundred common loci may confer susceptibility (Schizophrenia Working Group of the Psychiatric Genomics, 2014). Although individually common loci confer small amounts of risk, the combined effects of risk loci (including those that individually do not reach genome‐wide significance thresholds) can be investigated using risk profile scores (RPS). Schizophrenia RPS derived from large numbers of common loci now explain ∼7% of liability to schizophrenia (Schizophrenia Working Group of the Psychiatric Genomics, 2014).

Schizophrenia RPS models have been used to probe functional and structural neural networks associated with schizophrenia. Brain activation during working memory [Kauppi et al., 2015; Walton et al., 2014, 2013] and sentence completion [Whalley et al., 2015] was changed in the same direction in individuals with high schizophrenia RPS as in unaffected relatives of patients with schizophrenia. Conversely, most studies on associations between schizophrenia RPS and brain structure have been negative [Papiol et al., 2014; Van der Auwera et al., 2015]. No study to‐date has assessed the effects of schizophrenia RPS on reward‐based learning, an important and under‐explored component of psychopathology [see review by Forbes and Goodman, 2014]. Reward processing maps onto various valence systems defined by the Research Domain Objective Criteria (RDoC) for psychiatric research. Changes in reward processing are linked to chronic, mostly untreatable negative symptoms such as deficits in motivation and hedonic tone (https://www.nimh.nih.gov/research-priorities/rdoc/research-domain-criteria-matrix.shtml). In this study, we use summary data from schizophrenia PGC2 (Schizophrenia Working Group of the Psychiatric Genomics, 2014) to probe for differences in activation during a functional neuroimaging paradigm. Our study draws on data from the largest schizophrenia GWAS published to date to explore links between genetic schizophrenia risk and neural correlates of reversal learning. This task was chosen because a considerable body of evidence implicates alterations within a probabilistic learning network as part of the underpinning neurobiological pathology of schizophrenia.

Patients with schizophrenia show alterations within neural networks that support reward and learning processing during changing reward contingencies [Juckel et al., 2006; Koch et al., 2010; Rausch et al., 2014; Weiler et al., 2009]. It is suggested that such perturbations may reflect biological vulnerability (rather than a consequence of the disease, such as medication or disease chronicity) as they can also be seen in high‐risk and unmedicated patient groups [Esslinger et al., 2012; Juckel et al., 2012; Schlagenhauf et al., 2014; Rausch et al., 2015]. Considering that schizophrenia patients have observable deficits during probabilistic decision‐making and state‐independent alterations within neural networks in response to changing reward contingencies, we hypothesized that schizophrenia RPS will be associated with differences in brain function during a probabilistic learning task in healthy individuals.

We performed whole‐brain analysis, regressing individual schizophrenia RPS against individual brain activation maps for two functional contrasts maps during probabilistic decision‐making (a) shift > stay (assessing choice behavior) and (b) reward > punishment (assessing outcome behavior). Based on prior evidence, we expect to see an association between increased schizophrenia RPS and reduced blood oxygen level dependency (BOLD) signal in region of interests (ROIs) associated with reward processing. We therefore repeat these two regressions for a ROI analysis, restricting our search space to anatomical regions implicated in the (patho)physiology of probabilistic learning including the orbitofrontal cortex [Linke et al., 2012; Murray et al., 2008a; Tsuchida et al., 2010; Waltz and Gold, 2007], the anterior cingulate cortex [Culbreth et al., 2015; Koch et al., 2010; Nielsen et al., 2012; Strauss et al., 2014], striatum [Culbreth et al., 2015; Grimm et al., 2014; Mucci et al., 2015; Rausch et al., 2015; Schlagenhauf et al., 2014], and the hippocampus [Murray et al., 2008b; Wadehra et al., 2013]. We anticipate that schizophrenia RPS will be associated with altered activation within these regions during changes in reward contingencies.

## METHODS AND MATERIALS

### Participants

One hundred right‐handed Caucasian volunteers aged 19–47 were recruited from Cardiff University (staff and/or students). No participants reported any psychiatric illness [Goldberg, 1988] or use of psychotropic medication. After the study was described to the subjects, written informed consent was obtained. The study was approved by the ethics committee of the School of Psychology, Cardiff University. We had a final sample of 83 participants after removing individuals for whom failed quality control for genetic data (*n* = 10) or complete imaging data (*n* = 7) were not complete/available (see Table 1 for participant demographics). There were no significant associations between age, sex, schizophrenia RPS, and the behavioral parameters (*P* > 0.15, in all cases). There was no associations between schizophrenia RPS and age (*r* = 0.147, *P* = 0.185) and sex (*t* = −0.378, *P* = 0.707).

**Table 1:** Sample demographics and summary statistics for behavioral performance during probabilistic reversal learning task

	Demographic summary	Probabilistic learning performance (mean ± sd)	
Sample	*N* = 83	Accuracy 1^st^ reversal (%)	68.24 (20.20)
Age (mean ± sd)	23.95 (3.642)	Accuracy 1^st^ PE (%)	60.64 (29.63)
Sex	F = 49, M = 34	Accuracy 2^nd^ PE (%)	34.04 (28.84)
Schizophrenia RPS (range)	−6.12 × 10^−4^ (2 × 10^−4^)	Total earnings (pence)	0.23 (0.16)

Sample demographics for whole sample, after removing individual with missing genetic/imaging data (*n* = 83). HC, healthy controls; RPS, risk profile score; SD, standard deviation; PE, probabilistic error.

### DNA Extraction and Genotyping

Genomic DNA was obtained from saliva using Oragene OG‐500 saliva kits. Genotyping was performed using custom genotyping arrays (Illumina HumanCoreExome‐24 BeadChip) which contain 570,038 genetic variants (Illumina, Inc., San Diego, CA). Quality control was implemented in PLINK [Purcell et al., 2007], to ensure genotypes did not display ambiguous sex, cryptic relatedness up to third degree relatives by identity of descent, genotyping completeness <97%. We also removed non‐European ethnicity admixture detected as outliers in iterative EIGENSTRAT analyses of an LD‐pruned dataset [Price et al., 2006]. SNPs were excluded where the minor allele frequency was <1%, if the call rate <98% or if the χ^2^‐test for Hardy–Weinberg Equilibrium had a *P*‐value <1 e‐04. Individuals' genotypes were imputed using the pre‐phasing/imputation stepwise approach implemented in IMPUTE2/SHAPEIT with default parameters [Delaneau et al., 2012; Howie et al., 2009] and 1000 Genomes (December 2013, release 1000 Genomes haplotypes Phase I integrated variant set) as the reference dataset. Best guess genotypes were converted using gtool (http://www.well.ox.ac.uk/~cfreeman/software/gwas/gtool.html) and the default threshold of 0.8 was used.

### Generation of Risk Profile Scores

Schizophrenia RPS was calculated using the method described by the International Schizophrenia Consortium [International Schizophrenia et al., 2009]. Schizophrenia genetic risk was estimated using publicly available results data from an international GWAS of 34,241 schizophrenia cases and 45,604 controls (Schizophrenia Working Group of the Psychiatric Genomics Consortium). Single nucleotide polymorphisms (SNPs) were removed from the PGC‐schizophrenia GWAS data if they had a low MAF (minor allele frequency <0.01), and were subsequently pruned for linkage disequilibrium (*r* ^2^ < 0.2). This ensured all SNPs included in each schizophrenia RPS model were fairly independent. Schizophrenia RPS were calculated using the “score” command in PLINK which averages the number of risk alleles for each index SNP weighted by the natural logarithm of the SNP's odds ratio extracted from the PGC‐schizophrenia GWAS results. From the 7413342 SNPs, a total of 59,220 quasi‐independent SNPs were considered in the schizophrenia RPS (*P*_T_ < 0.5). We calculated schizophrenia RPS at the liberal *P*‐threshold (*P*_T_ < 0.5) because it has previously been used for schizophrenia RPS in neuroimaging studies [Whalley, et al., 2015, 2013]. Based on these prior studies, we hypothesize that *P*_T_ <0.5 will be most suitable to predict variability in probabilistic‐learning processing. There were no outliers in the schizophrenia RPS scores, which were normally distributed (Shapiro‐Wilk: *P* = 0.86). A power analysis performed in R (https://www.r-project.org/) using the “pwr” function [Champely, 2012] yielded 80% power to observe a small‐moderate effect (*r* = 0.3) of schizophrenia RPS on BOLD during probabilitsic learning (*n* = 83, α = 0.05, two‐sided).

### Probabilistic Decision‐Making Procedure

**Figure 1:**
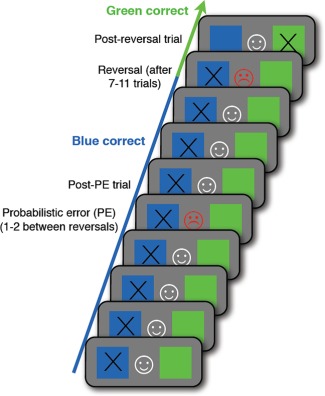
Probabilistic reversal‐learning paradigm. For each trial, two stimuli were presented. Participants selected a green or blue square and feedback was presented as a positive or negative emoticon. BOLD was modelled in post‐PE and post‐reversal trials, which reflected choice behavior (shift > stay; after rule reversal) or choice outcome (reward > punishment) under high levels of uncertainty.

Participants learned to choose one of two simultaneously presented colors (“blue” and “green”) by receiving monetary reward for correct choices and monetary punishment for wrong choices (e.g. +1 pence [p] for “blue” and −1p for “green”). After 7–11 trials, reward/punishment contingencies were reversed so that the previously rewarded color was now punished and vice versa. Participants were instructed to maximize their earnings during the learning session, which consisted of 12 reversal episodes in total (108 choice trials). Within each reversal episode we included either 1 or 2 PE (probabilistic error) trials, in which “wrong” feedback was given for correct choices, even though the reward contingencies had not changed. At the start of each choice trial, participants were presented with a response cue consisting of two white frames surrounding the colors and prompting the participants to press the left or right button on a response box to choose one color. Response feedback (choice outcome) was given subsequently using a centrally presented white “smiley” (correct choice) or red “frowny” (incorrect choice) face and an earnings counter changing incrementally by ± 1p. In trials following reversal or PE events, i.e., in those trials used for fMRI analysis (see below), response cues and feedback stimuli were presented with a jittered duration (cue: 4–8 s, mean 5.5 s; feedback: 0.75 s followed by 3–7 s [mean 4.5 s] ITI (Inter‐Trial‐Interval)). To reduce scanning time, in all other (standard) trials, we used fixed and shorter stimulus durations (cue: 2 s, feedback: 0.75 s). ITIs showed the two colors without response cue or feedback and were 0.5 s long after standard trials and between 4 and 8 s (mean 5.5 s) after PEs and reversals (see Fig. 1 for paradigm schematic).

### Behavioral Data Analysis

Overall learning performance was assessed as the accumulated earnings across all 108 choice trials. We also calculated trial‐based average accuracies (% choices corresponding to the correct color of each reversal episode) for the trials directly following PE and reversal events (post‐PE and post‐reversal trials) for each participant. Post‐PE and post‐reversal accuracy scores allowed us to measure impulsive choice behavior (high switch rates/low accuracies after PEs) and perseverative tendencies (low switch rates/low accuracies after reversals).

### Functional Image Processing

Gradient echoplanar imaging data was acquired for each subject using a 3T GT HDx system at CUBRIC (Cardiff University Brain Research Imaging Centre), School of Psychology, Cardiff University (parameters: 35 slices, slice thickness; 3 mm/1 mm gap, acquisition matrix; 64 × 64; FOV; 220 mm, TR 2000 ms, TE 35 ms, flip angle 90°, acceleration (ASSET) factor; 2). High‐resolution three‐dimensional T1‐weighted images were also acquired using a three‐dimensional FSPGR (fast spoiled gradient echo sequence) with 172 contiguous sagittal slices of 1 mm thickness (TR 7.9 s, TE 3.0 ms, TI 450 ms, flip angle 20°, FOV 256 × 256 × 176 mm, matrix size 256 × 256 × 192 to yield 1 mm isotropic voxel resolution images). Image processing and statistical analyses were conducted using statistical parametric mapping methods as implemented in FMRI Expert Analysis Tool (FEAT), Version 5.98, part of FMRIB's Software Library, http://www.fmrib.ox.ac.uk/fsl). The following prestatistics processing was applied; motion correction using MCFLIRT [Jenkinson et al., 2002]; slice‐timing correction using Fourier‐space time‐series phase‐shifting; non‐brain removal using BET [Brain Extraction Tool; Smith, 2002]; spatial smoothing using a Gaussian kernel of FWHM 5mm; grand‐mean intensity normalization of the entire 4D dataset by a single multiplicative factor; high‐pass temporal filtering (Gaussian‐weighted least‐squares straight line fitting, with sigma = 50.0  s). Registration to high‐resolution structural (single subject GLM (general linear model) and standard space (group‐level GLM) images was carried out using FLIRT [Jenkinson et al., 2002]. Time‐series analysis was carried out using FMRIB's Improved Linear Model (FILM) with local autocorrelation correction [Woolrich, et al., 2001]. None of the 83 participants had excessive head motion (0.09–1.68 mm, mean 0.38 mm). To further correct for any potential movement confounds, the 6 motion regressors estimated via MCFLIRT were added to the design matrix with automatic outlier deweighting. Group level analysis was carried out using FLAME [FMRIB's Local Analysis of Mixed Effects; Smith et al., 2004].

### fMRI Analysis

**Figure 2:**
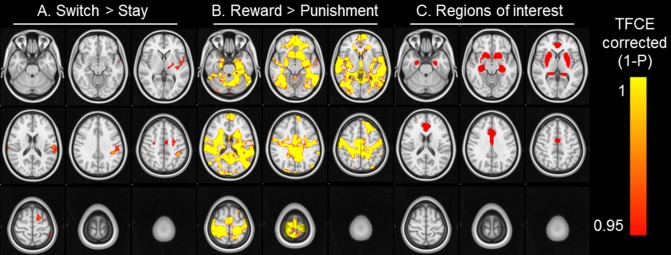
Right = right on all images. 1‐sample T‐tests for (a) shift > stay and (b) reward > punishment (corrected for multiple comparisons across the whole brain); P_FWE‐WHOLEBRAIN _< 0.05 using TFCE (threshold free cluster enhancement). We created a (c) region of interest (ROI) mask (binary) consisting of the bilateral orbitofrontal cortex, anterior cingulate cortex, nucleus accumbens, caudate, putamen, and hippocampus.

BOLD response analysis focused on brain activation differences as a function of (a) choice behavior (switch > stay response) and (b) choice outcomes (reward > punishment) in post‐PE and post‐reversal trials. No other contrasts were modelled in the study. We selected those trials for analysis as they yielded a comparatively balanced number of rewards/and punishments (correct/versus incorrect choices) and switch/stay responses, respectively, compared to standard trials (which were disproportionally more rewarded than punished and included more stay versus switch responses). These regressors modeled BOLD during decisional processes under high levels of uncertainty, i.e., after participants had to choose a stay or switch strategy in response to an unexpected punishment in the previous (PE or reversal) trial and during rewarding or punishment based feedback. BOLD signal changes were regressed by task predictor functions (switch > stay and reward > punishment) convolved with a canonical hemodynamic response function. For the switch–stay contrast, predictor functions were synchronized with the onset of the response cue in post‐PE/‐reversal trials; having a duration of 4000 ms and including both pre‐decisional and response processing. For the reward–punishment contrast and predictor time courses were locked to the onset of feedback stimuli in post‐PE and post‐reversal trials, with a fixed duration of 3750 ms, which corresponded to the earliest possible start of the next choice trial (see above). For each subject, statistical contrast images of shift > stay and reward > punishment were obtained. BOLD activity during probabilistic learning has shown good test–retest reliability [Freyer et al., 2009]. We then ran a multiple regression using the first‐level contrasts (switch > stay and reward > punishment) for each subjects co‐varying for schizophrenia RPS and potential confounds (age, sex, and ICV (intracranial volume)). To explore effects of schizophrenia RPS across the whole brain, we initially performed a whole‐brain‐based search on the two contrasts of interest. To follow up, we also performed ROI analysis on five merged anatomically defined regions (Harvard‐Oxford cortical and subcortical structural atlas) hypothesized to be associated with probabilistic learning and schizophrenia risk (see Fig. 2c). We corrected for the family‐wise error with nonparametric permutation testing (5000 permutations) and TFCE (threshold free cluster enhancement) which effectively controls the FWE (family wise error) rate (*P *< 0.05, corrected) [Nichols and Holmes, 2002; Smith et al., 2004]. Average parameter estimates for significant clusters (*P*_FWE_ < 0.05) were extracted for each individual using the fslmeants function.

## RESULTS

### Group‐Level Contrasts

We conducted two group‐wise one‐sample *t*‐tests to ascertain the functional networks that were recruited during (a) shift > stay and (b) reward > punishment (see Fig. 2a,b). After correcting of the FWE rate using TFCE (*P*_FWE_ < 0.05) across the whole brain, we found widespread activation in frontal (including the cingulate gyrus), temporal and limbic areas and the basal ganglia for the reward > punishment contrast, and in the superior frontal gyrus, motor cortex, and precuneus bilaterally and the left insula for behavioral switching, replicating findings from the literature [Linke et al., 2010].

### Schizophrenia RPS and Shift > Stay

**Figure 3:**
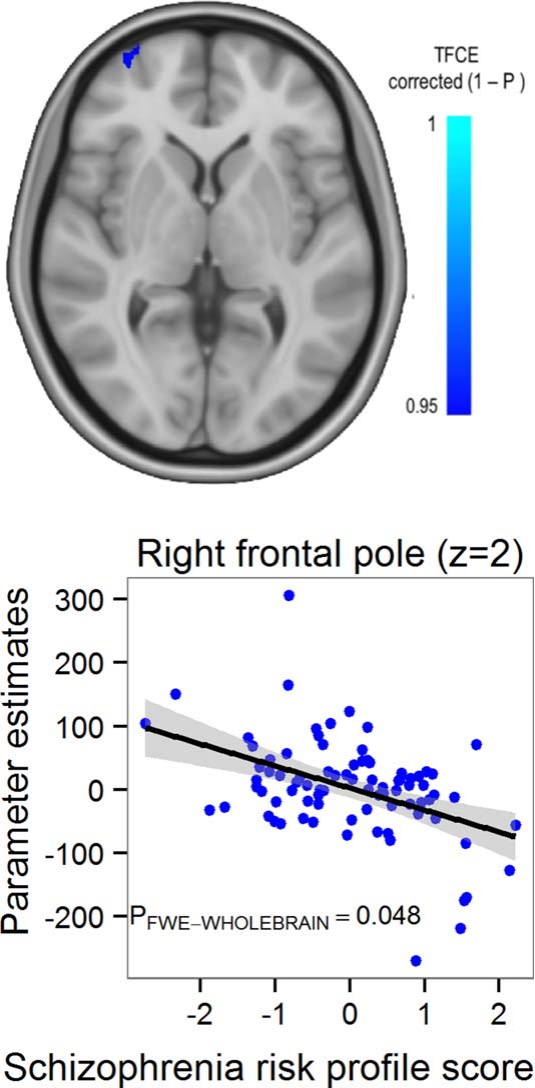
Right = right on all images. Whole‐brain analysis (*P* < 0.05, corrected across whole brain) revealed a negative association between schizophrenia RPS and the right frontal pole. Partial correlations controlling for age, sex, and ICV, as well as the removal of BOLD outliers (as defined by ±2.5 SDs) did not significantly the association between schizophrenia RPS. Grey shadow represents 95% confidence interval of the regression slope. Schizophrenia RPS is *Z*‐normalized for illustration purposes.

**Figure 4:**
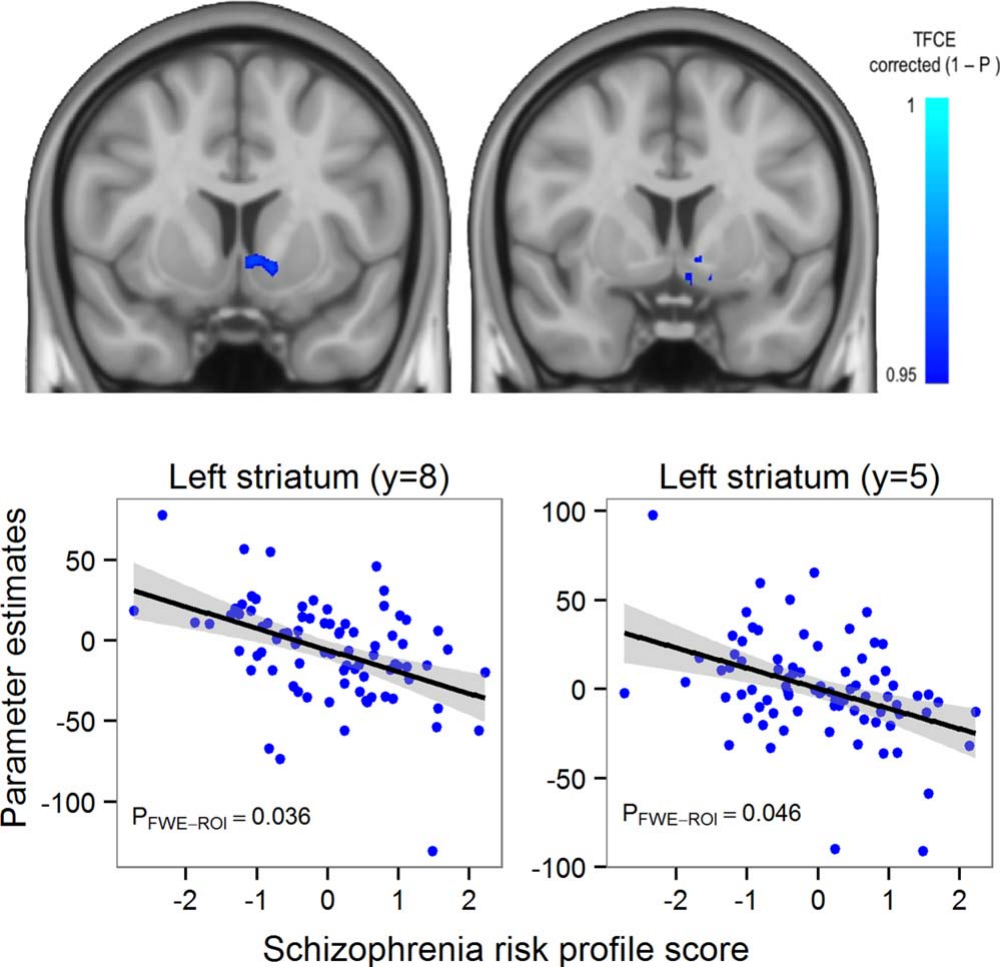
Right = right on all images. Region of interest analysis (*P* < 0.05, corrected across ROI mask) revealed a negative association between schizophrenia RPS and two clusters in the left ventral striatum. The cluster within the ventral striatum (*y* = 5) is the cluster in the bottom left of right‐hand image; the other two are part of the cluster presented in the image on the left. Partial correlations controlling for age, sex, and ICV, as well as the removal of BOLD outliers (as defined by ±2.5 SDs) did not significantly affect any of the associations between schizophrenia RPS. Grey shadow represents 95% confidence interval of the regression slope. Schizophrenia RPS is *Z*‐normalized for illustration purposes.

In the whole‐brain analysis, we found a significant negative association with schizophrenia RPS during shift > stay in the right frontal pole (P_FWE‐WHOLEBRAIN _= 0.037; see Fig. 3). We found no positive whole‐brain associations with schizophrenia RPS during shift > stay (*P*_UNCORRECTED_ = 0.019) and in the ROI mask (*P*_UNCORRECTED_ = 0.037). In the ROI analysis, we found additional negative associations between schizophrenia RPS during shift > stay in the left ventral striatum (*P*_FWE‐ROI_ = 0.036 & 0.046; see Fig. 4). For MNI (Montreal Neurological Institute) coordinates of significant clusters, see Table 2.

**Table 2:** Significant clusters for whole‐brain and ROI schizophrenia RPS analysis (shift > stay)

Whole brain analysis	*k*	X	Y	Z	*P*_FWE‐WHOLE BRAIN_
Right frontal pole	33	34	58	0	0.048
Region of interest analysis					*P*_FWE‐ROI_
Left ventral striatum	44	−4	6	−12	0.036
Left ventral striatum	5	−4	4	−12	0.046

Results from whole brain analysis and ROI analysis (shift > stay; following rule reversal). *k* = number of continuous voxels. Coordinates (*X*, *Y*, *Z*) are in MNI (Montreal Neurological Institute) space. Results corrected form multiple comparisons across the whole brain (*P*_FWE‐WHOLEBRAIN_ < 0.05) or across the region of interest (*P*_FWE‐ROI_ < 0.05).

### Schizophrenia RPS and Reward > Punishment

We found no significant whole‐brain effects on schizophrenia RPS during reward > punishment in (*P*_UNCORRECTED_ = 0.004: highest peak). We found no significant effects in the ROI mask on schizophrenia RPS during reward > punishment, although there was a trend toward a positive association between schizophrenia RPS and BOLD in the right hippocampus (*P*_UNCORRECTED_ = 0.0002; *P*_FWE‐WHOLEBAIN_ = 0.169; highest peak).

### Associations Between BOLD and Probabilistic Learning Behavior

Schizophrenia RPS did not predict any of the behavioral parameters (accuracy after first probabilistic error, accuracy after first reversal, total earnings, *P* > 0.1 in all cases). We proceed to explore the functional role of significant clusters. Post‐hoc analysis confirmed that age, sex, and ICV were not associated with BOLD in the clusters identified (*P *> 0.5, in all cases). We proceeded to correlate behavioral performance with parameter estimates derived from our significant clusters (identified after correction for family wise error rate across either the whole brain or across an ROI (*P*_FWE_ < 0.05. Parameters estimates from whole‐brain clusters (*n* = 1) and ROI clusters (*n* = 2) were not associated with any behavioral parameters (*P* > 0.5 in all cases).

## DISCUSSION

We observed an association between schizophrenia RPS and BOLD in healthy participants during probabilistic learning. Specifically, we observed a negative correlation between RPS and BOLD signal during choice processing in the right frontal pole (whole‐brain analysis) and the left ventral striatum (ROI analysis). As far as we are aware, this is the (a) first functional neuroimaging study to use schizophrenia PGC2 as training data and (b) the first polygenic imaging study to assess the effects of schizophrenia RPS on reward and/or learning processes. The former point is important as using summary statistics from larger data sets such as schizophrenia PGC2 (Schizophrenia Working Group of the Psychiatric Genomics, 2014) enhances the power and predictive capacity in discovery samples [Dudbridge, 2013].

Dysfunction in frontostriatal circuitry may be a causal mechanism that links the deficits in goal‐directed and motivated behavior that schizophrenia patients frequently experience to negative clinical symptoms [Barch and Dowd, 2010]. We propose these changes may reflect a genetic susceptibility mechanism that underpin motivational aspects of reinforcement learning or reduced cognitive control [Culbreth et al., 2015; Waltz et al., 2013], which could lead to impaired learning of shifting reward contingencies. While some studies observe hyperactivity in striatal regions in schizophrenia patients and their relatives during reward anticipation [Esslinger et al., 2012; Grimm et al., 2014], our hypothesis is supported by observations that the reduced ventral striatum activation that schizophrenia patients display during reward processing and set‐shifting may be related to negative clinical symptoms such as deficits in motivation and affect [Waltz et al., 2013, Arrondo et al., 2015; Kirschner et al., 2015; Simon et al., 2015; Subramaniam et al., 2015]. The association between attenuated BOLD in this frontostriatal circuit and negative symptoms has also been observed in individual with familial genetic risk [de Leeuw et al., 2015]. Alterations in frontostriatal BOLD may provide a mechanistic explanation for the observation that patients with schizophrenia show disruptions in set‐shifting ability (perseveration) following rule reversal [Strauss et al., 2014; Waltz et al., 2013].

Our results also conform to an emerging hypothesis suggesting that frontostriatal dysfunction during probabilistic learning may be a core neurobiological component of schizophrenia pathophysiology [Murray et al., 2008b; Rausch et al., 2014, 2015; Schlagenhauf et al., 2014]. We found schizophrenia RPS‐related clusters in (a) cortical regions previously implicated in reward/reversal‐learning processes such as the frontal pole [Burke et al., 2013; Ramnani et al., 2004] and (b) regions hypothesized to mediate schizophrenia‐related deficits during reversal learning such as the ventral striatum [Gold et al., 2008; Waltz and Gold, 2007].

A growing body of work suggest that schizophrenia RPS is associated with functional changes similar to the aberrant patterns of activation seen in individuals with familial risk for schizophrenia [Kauppi et al., 2014; Walton et al., 2014, 2013; Whalley et al., 2015]. We build on this work by assessing how cumulative genetic susceptibility for schizophrenia may affect parameters of reward and/or learning. Taken together, these studies suggest that the alterations in BOLD during reward function observed in familial risk for schizophrenia may be explained partly by common genetic variants. Candidate schizophrenia genes such as *DRD2* and *CACNA1C* have also been shown to affect reward‐based learning and BOLD correlates [Jocham et al., 2009; Klein et al., 2007; Lancaster et al., 2014; Wessa et al., 2010]. Although candidate risk variants may only explain small amounts of risk (Schizophrenia Working Group of the Psychiatric Genomics, 2014), they support a broader hypothesis that common genetic variation may contribute to core neurobiological features of schizophrenia. Candidate studies also provide plausible neurobiological mechanisms by which schizophrenia risk variants confer susceptibility. Further polygenic pathway analysis will be required to assess the cumulative effects of risk variants on functionally annotated pathways [Chen et al., 2015]. Moreover, this study supports a growing body of work suggesting that schizophrenia RPS models are associated with core neurobiological phenotypes that are associated with psychiatric illness [Kauppi et al., 2015; Walton et al., 2014, 2013; Whalley et al., 2012; Whalley et al., 2015]. Our sample size was modest for a genetic neuroimaging study, and replication studies are needed to determine the size and nature of the effects of schizophrenia RPS on the reward system. However, the utility of the updated schizophrenia RPS should add considerable power to this investigation, compared to studies of single candidate SNPs, each of which contributes only a very small amount to disease liability [Dima and Breen, 2015; Dudbridge, 2013].

## CONCLUSIONS

Polygenic imaging investigations are essential for understanding how increased common genetic risk for schizophrenia may affect brain function. We found associations between schizophrenia RPS with frontostriatal activation during choice behavior (shift > stay), but not outcome processing (reward > punishment) during probabilistic reversal learning. In conclusion, we provide evidence suggesting that alterations in neural networks supporting probabilistic learning may reflect the genetic risk for schizophrenia. We found associations between schizophrenia RPS and probabilistic learning processing in regions previously shown to be disrupted in schizophrenia and in regions where BOLD may reflect negative symptoms such as motivation [Subramaniam et al., 2015], anhedonia [Waltz et al., 2013], and apathy [Simon et al., 2015]. Further studies will be required to assess the neurobiological pathways that contribute to polygenic effects of schizophrenia risk on neural activation and explore whether other reward and/or learning phenotypes are also associated with risk for schizophrenia. Our findings thus support the validity of the novel schizophrenia RPS approach for the understanding of the behavioral and neural pathways through which neuropsychiatric risk genes contribute to clinical phenotypes, which is one of the central questions of translational neuroscience today.
